# Prognostic value of hematological parameters in patients with acute myocardial infarction: Intrahospital outcomes

**DOI:** 10.1371/journal.pone.0194897

**Published:** 2018-04-18

**Authors:** José Gildo de Moura Monteiro Júnior, Dilênia de Oliveira Cipriano Torres, Maria Cleide Freire Clementino da Silva, Cyntia Maria de Holanda Martins, Izadora Karina da Silva, Monique Evelyn Mendonça do Nascimento, Ana Célia Oliveira dos Santos, Ulisses Ramos Montarroyos, Dário Celestino Sobral Filho

**Affiliations:** 1 Coronary Care Unit of PROCAPE (Pernambuco Cardiac Emergency Hospital), University of Pernambuco (UPE), Recife, Pernambuco, Brazil; 2 Laboratory of PROCAPE, University of Pernambuco, Recife, Pernambuco, Brazil; 3 University of Pernambuco, Recife, Pernambuco, Brazil; University of Tampere, FINLAND

## Abstract

**Background:**

The intensity of the inflammatory response and hemodynamic repercussion in acute myocardial infarction causing the presence in the peripheral circulation of nucleated red blood cells (NRBCs), increases in mean platelet volume (MPV) and neutrophil to lymphocyte ratio (NLR) are associated with a poorer prognosis. The aim of this study was to assess the role of these hematological biomarkers as predictors of all causes of mortality during the hospitalization of patients with acute myocardial infarction.

**Methods:**

Nucleated red blood cells, mean platelet volume and neutrophil to lymphocyte ratio were measured daily during the hospitalization of the patients with acute myocardial infarction. We excluded patients younger than 18 years, on glucocorticoid therapy, with cancer or hematological diseases and those that were readmitted after hospital discharge. We performed a multiple logistic analysis to identify independent predictors of mortality.

**Results:**

We included 466 patients (mean age 64.2 ± 12.8 years, 61.6% male). The prevalence of NRBCs in the sample was 9.1% (42 patients), with levels > 200/μL in 27 patients (5.8%). The mean MPV value was 10.9 ±0,9 and the mean NLR value was 3.71 (2,38; 5,72). In a multivariate analysis of serum NRBCs (HR 2.42, 95% CI: 1.35–4.36, p = 0.003), MPV (HR 2.97, 95% CI: 1.15–7.67, p = 0.024) and NLR (HR 5.02, 95% CI: 1.68–15.0, p = 0.004). The presence in the peripheral blood of NRBCs, increased in mean platelet volume and neutrophil to lymphocyte ratio were associated with higher mortality.

**Conclusions:**

Nucleated red blood cells, mean platelet volume and neutrophil to lymphocyte ratio are independent predictors of intrahospital mortality. Therefore, an important tool in intrahospital clinical surveillance.

## Introduction

Acute myocardial infarction (AMI) is a frequent emergency in the world, with great potential for morbidity and mortality despite all advances in treatment in the last three decades [1]. It is an essentially inflammatory disease and, depends on the extent of cardiac damage, with hemodynamic repercussion. These inflammatory and hypoxemic processes have been associated with the presence of hematological markers in the peripheral blood owing to the high concentrations of erythropoietin, interleukin-3 and interleukin-6 caused by local or systemic disorders in critical cardiac patients [2].

Researches have shown that there is an increase in myeloid activity in AMI and it has arisen strong interest in hematological parameters, given that they may provide independent information on pathophysiology and risk stratification [3]. Therefore, the study of the complete blood count is of great importance in the acute coronary syndrome with, for examples, red cell distribution width (RDW) is a measure of variations in the volume of red blood cells and it is an essential predictor of severity coronary artery disease among patients with AMI [4,5], white blood cell count (WBC) elevated was found to be a relevant death risk factor during the first 30 days and 6 months following the myocardial infarction [6] and platelet distribution width (PDW) indicates a varied size of platelets and it also serves as a useful prognostic factor for long-term mortality in patients after AMI [7,8]. Mean platelet volume (MPV) is an indicator of platelet size and activation marker [9–15]. In the recent years, numerous papers have been published regarding the value of platelet to lymphocyte ratio (PLR) [16–19], mean platelet volume to lymphocyte ratio (MPVLR) [20,21] and neutrophil to lymphocyte ratio (NLR) [22–36] in predicting short and long-term mortality in patients with ST-elevation (STEMI) and with non-ST elevation (non-STEMI). The lymphocyte count is inversely association with inflammation and, low lymphocyte count is a poorer prognostic marker in patients with AMI [37].

Nucleated red blood cells (NRBCs) are immature erythrocyte cells present in the bone marrow in the process of hematopoiesis. In healthy adult, there aren’t NRBCs in the peripheral blood. Prior studies have shown that severe hypoxemia or inflammation are responsible for the presence of NRBCs in peripheral blood, when hematological and oncological diseases are excluded. Therefore, the presence of NRBCs in the peripheral circulation are associated with poorer prognosis [38–40]. The scientific evidences have demonstrated the value of this variable with importance tool in the intrahospital surveillance.

In this study, despite of the diversity of hematological variables, all components of the hemogram were represented: NRBCs (red blood cells), NLR (white blood cells) and MPV (platelets). Therefore, the aim of this study was to assess the role of these hematological biomarkers as predictors of all causes of mortality during the hospitalization of patients with acute myocardial infarction.

## Materials and methods

### Subjects and protocol

This study was approved by the Research Ethics Committee in the HOSPITAL COMPLEX HUOC/PROCAPE of the University of Pernambuco under number CAAE: 51802115.7.0000.5192 (Brazil Plataform). All consecutive patients admitted with acute myocardial infarction to the Pernambuco Cardiac Emergency Unit (PROCAPE), a specialized tertiary care cardiovascular teaching hospital with 250 beds, between January 2016 and September 2016 were included in the research. We excluded patients younger than 18 years, on glucocorticoid therapy, with câncer or hematological diseases and those that were readmitted after hospital discharge. All patients included in the study signed a free and informed consent form.

The diagnosis of acute myocardial infarction was given by clinical history, electrocardiography and laboratory abnormality (troponin). After the diagnosis was confirmed, the patient or family member answered about risk factors. He was simply asked if he was taking any medication for high blood pressure, diabetes mellitus, dyslipidemia and depression. Patient with arterial blood pressure measurements above 140/90 mmHg was considered hypertensive and fasting plasma glicose above 126 mg/dL was considered diabetes mellitus. He was also asked about sedentary lifestyle, smoking, kidney disease, and family history of coronary artery disease. Then, this patient would follow the protocol of the institution until discharge but, only suggested in a previous communication to the medical staff of the hospital, the request for a daily blood count.

In the first twenty-four hours of the admission, the patients were classified in the AMI with ST segment elevation (STEMI)) and AMI without ST segment elevation(non-STEMI), and their respective Killip and TIMI Risk risk scores were calculated.

### Laboratory tests

Blood samples were obtained daily in the morning until discharge from the hospital. Complete blood count parameters including NRBCs, leukocytes, neutrophils, lymphocyte, platelet, MPV were measured using a Sysmex XE-2100 blood analyzer (Sysmex, Kobe, Japan) [41,42]. The troponin samples, as well as other biochemical measurements and electrolyte levels, were measured using a Roche Cobas Integra 400 analizer with a reference value of 0.014 μg / L.

A positive NRBC was defined as any value above zero, high MPV cut-off level is ≥10.4 fL and NLR was calculet by dividing the neutrophil count with the lymphocyte count with high cut-off level is ≥ 3.7.

### Statistical analysis

Contínuos variables were expressed as mean ± standard deviation or median and quartiles, as appropriated analyze. Categorical variables were presented as absolute values and percents. Categorical variables were compared using two-tailed Pearson’s chi-squared (X^2^) test with the Yates correlation or Fisher’s exact test. The comparison of means, to establish the normality of the distribution, was carried out using the Kolmogov-Smirnov test, followed by Student’s t test for normal distribution variables or Mann-Whitney’s non-parametric test form non-normal distribution variables. The relative mortality risk was calculated for clinical and laboratory variables, with confidence intervals of 95%. Logistic univariate regressions were performed to evaluate predictors of mortality of these variables. A multivariate logistic regression model was used to identify independent predictors of mortality. Variables with p <0.05 on univariate analysis were entered a multivariate analysis. Due to the highly skewed distribution of the NRBC, we chose to perform its analysis as a binary variable based on the presence or absence of NRBC in the peripheral blood.

The level of statistical significance adopted was p < 0.05. Sample size was calculated to assess a mortality Hazard Ratio between patients with AMI and NRBCs, MPV and NLR of according to previous study, assuming an α-error of 5% and a statistical power of 95%. The minimum sample size was 170 patients. Statistical analyses were conducted using the Statistical Program for Social Sciences (SPSS), version 10.0 for Windows.

## Results

**Fig 1:**
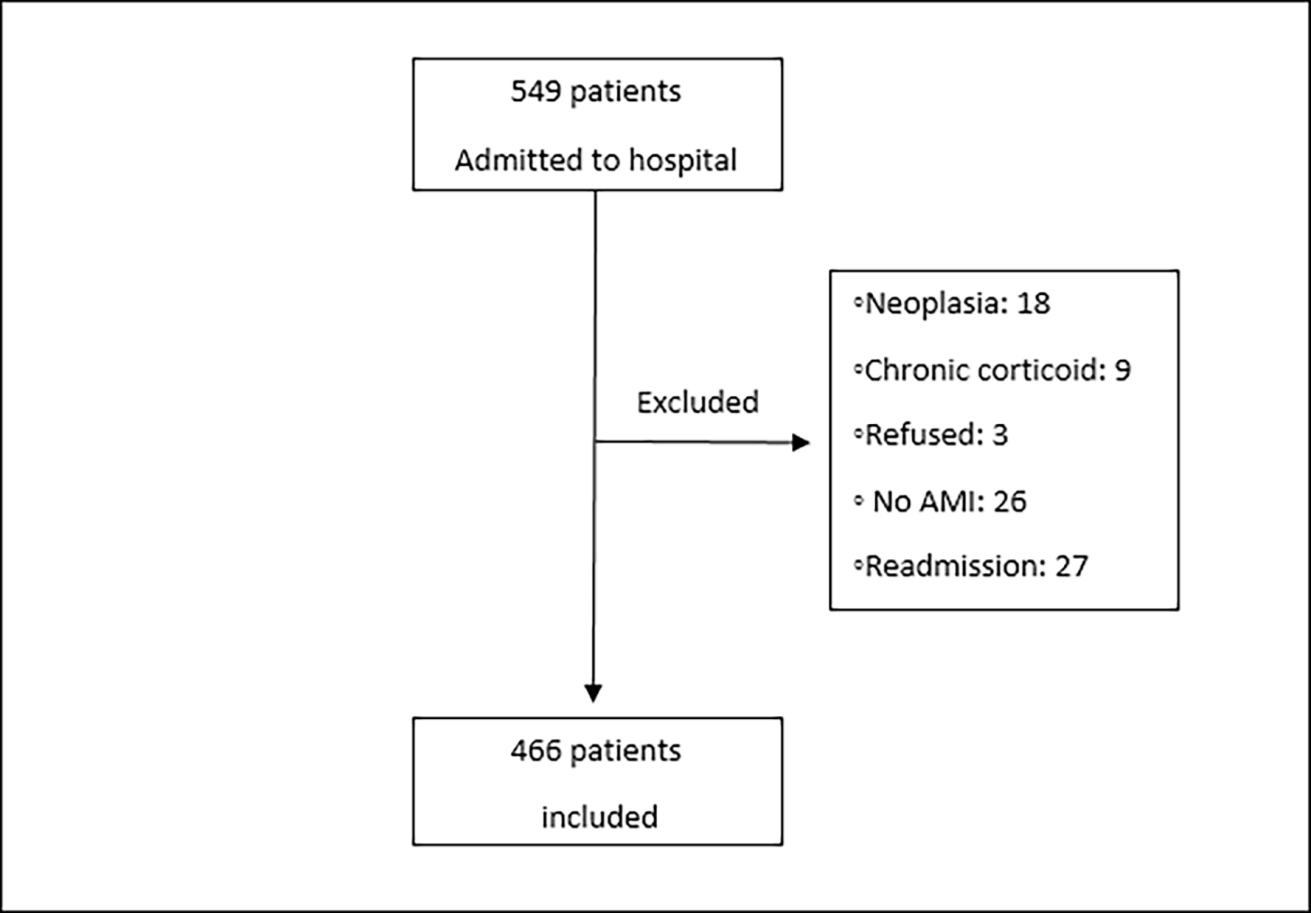
Flowchart of patients.

A total of 549 patients initially screened, of whom 83 were excluded as shown in Fig 1. There were no follow-up losses and the final sample comprised 466 patients (mean age 64.2 ± 12.8 years, 61.6% male) and AMI with STEMI (70%). The demographic, clinical and laboratory characteristics of the study patients are described in Table 1. A total of 27 cardiac surgeries (5.8%), 226 coronary angioplasties (48.5%) were performed during hospitalization of these patients. Among all patients, 32 (6.9%) had used antibiotic therapy. The total mortality of the study was 11.8% (55 patients).

**Table 1:** Characteristics of patients with acute myocardial infarction.

Characteristics	Statistics
**Age (mean ± SD)**	64.2 ± 12.8
**Gender: Male**	287 (61.6%)
**Risk factors**	
**Systemic arterial hypertension**	335 (71.9%)
**Diabetes mellitus**	173 (37.1%)
**Kidney disease**	38 (8.2%)
**Family history of coronary artery disease**	220 (47.2%)
**Dyslipidemia**	178 (38.2%)
**Depression**	50 (10.7%)
**Smoking**	194 (41.6%)
**Sedentary lifestyle**	232 (49.8%)
**Acute myocardial infarction**	
**STEMI**	326 (70.0%)
**Killip score**	
**Killip I and II (low risk)**	298 (91.4%)
**Killip III and IV (high risk)**	28 (8.6%)
**Non-STEMI**	140 (30.0%)
**TIMI Risk**	
**0 to 3 (low risk)**	40 (28.9%)
**4 to 7 (high risk)**	98 (71.1%)
**Red cells**	4.40 ± 0.62
**Hemoglobin**	13.0 ± 2.00
**Hematocrit**	38.5 ± 5.22
**NRBC**	
**Presence (≥1)**	42 (9.1%)
**Absence (0)**	421 (89.9%)
**NRBC Maximum**	
**Zero**	421 (89.9%)
**1 to 100**	10 (2.2%)
**101 to 200**	5 (1.1%)
**> 200**	27 (5.8%)
**Leukocytes**	10.5 (8.4, 12.8)
**NLR**	3.71 (2.38, 5.72)
**CRP**	36.7 (11.6, 86.6)
**Platelets**	231 (195.7,278)
**MPV**	10.9 ± 0.9
**IG%**	0.3 (0.22, 0.45)
**TNT**	1.87 (0.44, 4.39)
**RDW SD**	43.2 (41.1, 45.4)
**RDW CV**	13.5 (12.9, 14.2)

Abbreviations: STEMI: with ST elevation myocardial infarction; non-STEMI: with non-ST elevation myocardial

Infarction; NRBC: nucleated red blood cells; NLR: neutrophil to lymphocyte ratio; CRP: C-reactive protein

MPV: mean platelet volume; IG: immature granulocytes; TNT: troponin T; RDW SD: Red Blood Cell Distribution

Width measured by Standard Deviation; RDW CV: Red Blood Cell Distribution Width measured by Variation Coefficient

**Fig 2:**
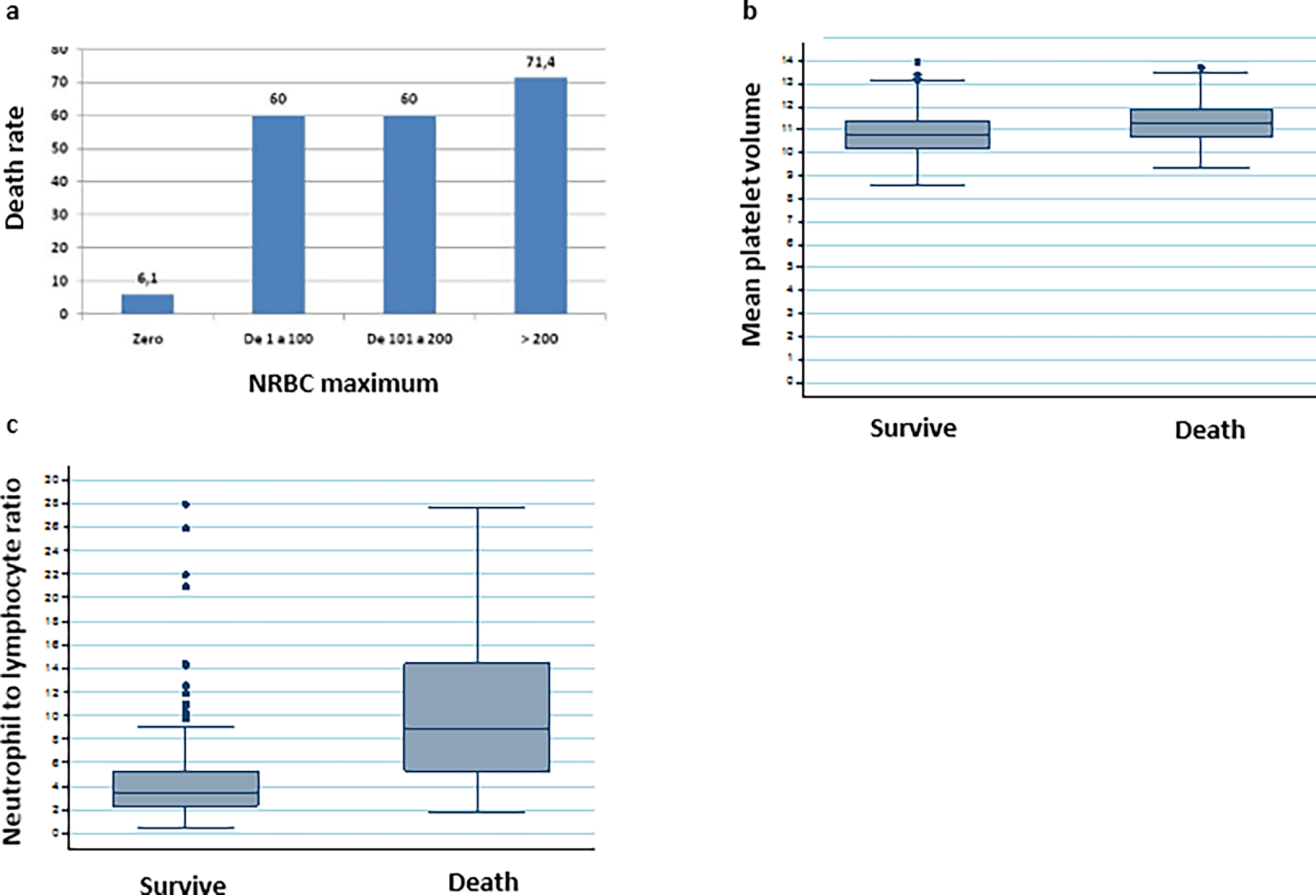
a. Nucleated red blood cells, b. Mean platelet volume, c. Neutrophil to lymphocyte ratio and intrahospital mortality.

The presence of NRBCs in the sample was 9.1% (42 patients), with levels > 200/μL in 27 patients (5.8%). The mean MPV value was 10.9 ±0,9 and the mean NLR value was 3.71 (2,38; 5,72) (Table 1). The intrahospital mortality is associated with presence of NRBCs in peripherical blood and increases in mean MPV and NLR (Fig 2).

The factors associated with intrahospital mortality in this study are the age (HR 2.52, 95% CI: 1.37–6.65, p = 0.003), NRBC (HR 5.65, 95% IC: 3.23–9.88, p<0.001), MPV (HR 4.46, 95% IC: 1.78–11.2, p = 0.001) and NLR (HR 11.3, 95% IC: 4.06–31.2, p = 0.000). The age among the demographic characteristics is significantly associated with mortality (HR 2.52, 95% CI: 1.37–6.65, p = 0.003). However, in this sample there is an inverse association with family history (HR 0.35, 95% CI: 0.19–0.67, p = 0.002) (Table 2).

**Table 2:** Factors related to intrahospital mortality among patients with acute myocardial infarction.

Factors	Death of numbers	Death Risk	
		HR (95% CI)	p-value
**Age**			
**<65 years**	14	Reference	-
**≥ 65 years**	41	2.52 (1.37–6.65)	0.003
**Sex**			
**Female**	21	Reference	-
**Male**	34	1.20 (0.69–2.09)	0.517
**Risk factors**			
**Systemic arterial** **hypertension**	45	1.48 (0.75–2.95)	0.261
**Diabetes mellitus**	27	1.41 (0.82–2.40)	0.210
**Kidney disease**	5	1.01 (0.40–2.52)	0.991
**Family history of** **coronary disease**	12	0.35 (0.19–0.67)	0.002
**Dyslipidemia**	18	0.64 (0.36–1.13)	0.124
**Depression**	1	0.19 (0.03–1.36)	0.098
**Smoking**	20	1.03 (0.59–1.80)	0.911
**Sedentary lifestyle**	32	1.23 (0.72–2.12)	0.442
**Laboratory Measures**			
**Erythrocyte^**a**^**	-	0.41 (0.25–0.68)	0.001
**Hemoglobin^**a**^**	-	0.83 (0.71–0.98)	0.024
**Hematocrit^**a**^**	-	0.93 (0.87–0.98)	0.018
**Leukocytes (>10.5)^**b**^**	45	4.57 (2.30–9.09)	0.000
**CRP (>36.7)^**b**^**	41	3.55 (0.46–3.88)	0.002
**Platelets (>231)^**b**^**	8	6.93 (3.21–14.9)	0.000
**IG% (>0.3)^**b**^**	49	6.09 (2.59–14.3)	0.000
**TNT (>1.87)^**b**^**	37	3.03 (1.69–5.42)	0.000
**RDW SD (>43.2)^**b**^**	43	3.56 (1.83–6.92)	0.000
**RDW CV (>13.5)^**b**^**	43	3.15 (1.61–6.12)	0.001
**NLR:**			
**<3.7**	4	Reference	-
**≥ 3.7**	51	11.3 (4.06–31.2)	0.000
**NRBC:**			
**Absence (0)**	26	Reference	-
**Presence (≥1)**	29	5.65 (3.23–9.88)	<0.001
**MVP:**			
**<10,4**	6	Reference	-
**≥ 10.4**	49	4.46 (1.78–11.2)	0.001

^a^ Decreased risk with the increase of one unit of the laboratory marker

^b^ Risk for values above the median

**Fig 3:**
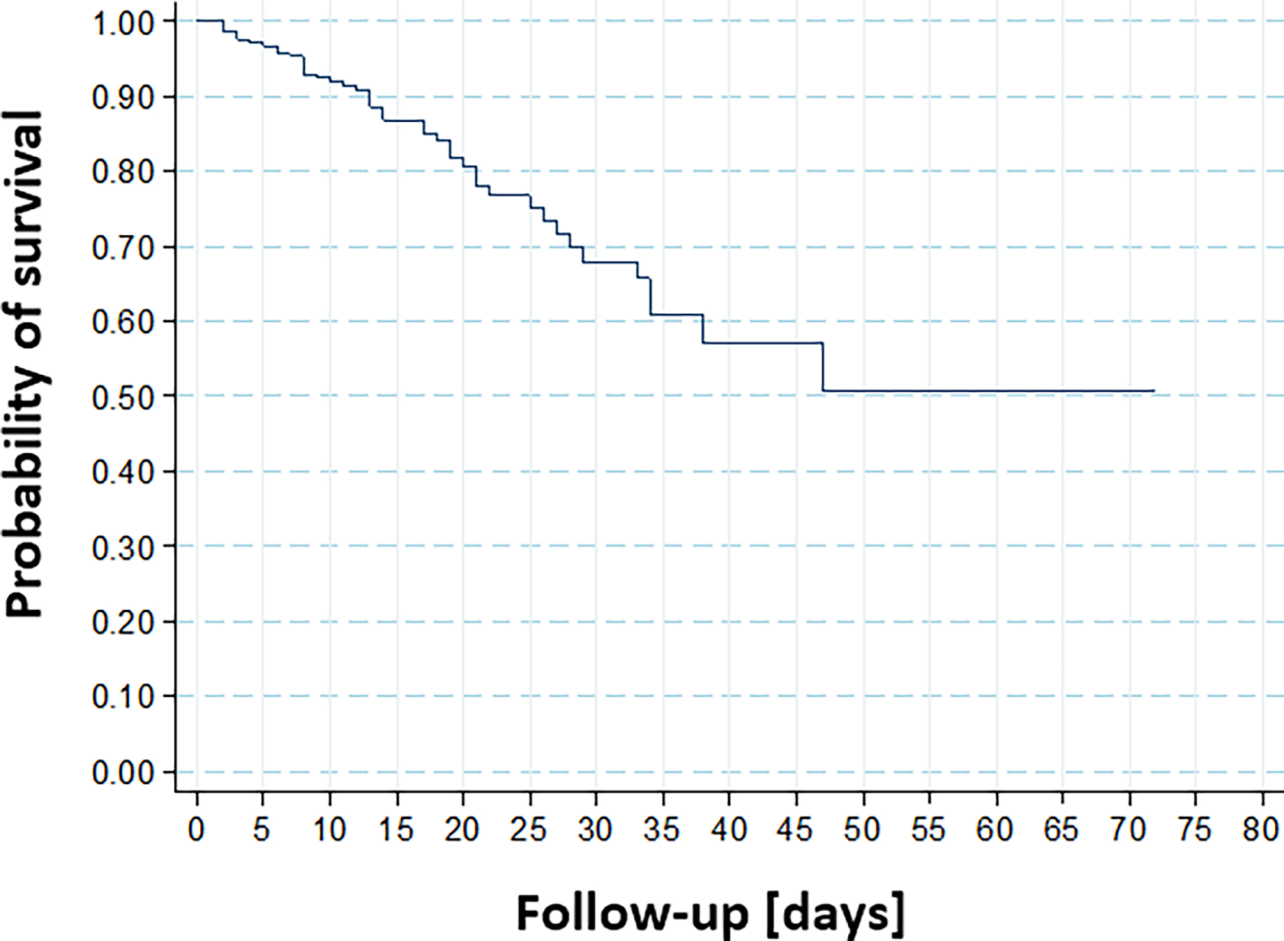
Kaplan-Meyer curve of intrahospital mortality.

After adjustment, multivariate analysis of the factors associated with intrahospital mortality among patients with acute myocardial infarction as described in Table 3. The survival curve of intrahospital mortality is shown in Fig 3.

**Table 3:** Multivariate analysis of factors related to intrahospital mortality among patients with acute myocardial infarction.

Factors	Death Risk	
	HR (95% CI)	p-value
**Age**		
**<65 years**	Reference	-
**≥ 65 years**	1.88 (1.02–3.49)	0.043
**Laboratory Measures**		
**Leukocytes (> 10.5)^**a**^**	2.01 (0.97–4.17)	0.059
**TNT (> 1.87)^**a**^**	1.76 (0.98–3.16)	0.057
**NLR:**		
**<3.7**	Reference	-
**≥ 3.7**	5.02 (1.68–15.0)	0.004
**NRBC:**		
**Absence (0)**	Reference	-
**Presence (≥ 1)**	2.42 (1.35–4.36)	0.003
**MVP:**		
**<10,4**	Reference	-
**≥ 10.4**	2.97 (1.15–7.67)	0.024

^a^ Risk for values above the median

## Discussion

In this study, the main finding was that the daily quantifications of NRBCs, MPV and NLR are important to the prognosis in intrahospital patients with AMI. These variables reflect the severity of systemic impairment and consequently on clinical outcome at each moment, depending on the response to the proposed treatment. It is linked to an increased myeloid activity, inducing the release of hematopoietic stem cells from bone marrow niches and causing the further systemic stimulation of atherosclerotic plaques [3]. However, there are also other co-morbidities that may contribute to the clinical state and consequently the time of intrahospital stay and mortality. Therefore, the intensity of inflammation and hypoxemia of multifactorial origin during the hospitalization of these patients causes this hematopoietic feedback.

The presence of NRBCs in peripheral blood in patients with AMI, as demonstrated in this study, are associated with mortality (HR 2.42, 95% CI: 1.35–4.36, p = 0.003). These findings are in accordance with recent published papers, although in difference populations. Stachon et al. [38] studied this variable in the intensive care unit and their conclusion were this parameter may serve as a daily indicator of patients at high mortality risk and they should not be relocated to a normal ward while remaining NRBC-positive. Desai et al. [39] associated NRBC-positive with a higher mortality rate in patients with surgical sepsis (27% vs 12%, p = 0.007). Kurt et al. [40] associated the NRBCs with arterial oxygen partial tension (prior to the initial detection of NRBCs in the peripheral blood, pO_2_ levels were significantly lower in patients who died than in surviving patients).

In this same inflammatory and hypoxemic context due to several etiological factors, as observed in the present study, the increased MPV are also associated with mortality in patients with AMI (HR 2.97, 95% CI: 1.15–7.67, p = 0.024). Previous papers share the information that the larger platelets are metabolically and enzymatically more active than small platelets and are reflected by elevation in MPV [12,13]. Therefore, these biological variables that determine important role in the development of intravascular thrombus [11]. Hilal et al. [9] employed the same cut-off value of 10.4 in their paper in which they demonstrated that MPV ≥ 10.4 is a predictor of severe atherosclerosis with sensibility of 39% ans specificity of 90% (ROC curve: 0.631, 95% CI: 0.549–0.708, p = 0.003).

The blood count is performed on admission and daily routinely and carries important prognosis information. Our findings in this study met high association between NLR and intrahospital mortality in patients with AMI (HR 11.3, 95% CI: 4.06–31.2), p = 0.000). Studies have shown that the NLR is a strong predictor of short and long-term mortality in stable and unstable coronary insufficiency [22]. It is relationship with the inflammation, which participates articulately in all stages, from beginning of the lesion to the progression and destabilization of the plaque [35]. There are studies comparing NLR with SYNTAX score which evaluated the severity coronary angiographic, with GRACE, KILLY and TIMI risk scores which evaluated demographic data and variables that determine the intrahospital death in patients with AMI [24,28]. Therefore, NLR may be used as cost-effective predictors of inflammation and AMI complications.

The selection of patients from single center may raise a limitation. However, we sought to increase the sample to minimize possible biases and confounding factors in the study.

## Conclusion

The survival rate of patients with acute myocardial infarction decreases after hospital stay due to several complications that may occur during this period. Therefore, objective monitoring tools are needed.

Nucleated red blood cells, mean platelet volume and neutrophil to lymphocyte ratio are independent markers of death, express the degree of systemic inflammation and hypoxemic impairment, which are the major pathophysiological mechanisms of diseases. Therefore, an important tool in intrahospital clinical surveillance. However, more research on these hematological biomarkers and dissemination of knowledge in the daily practice of the multiprofessional hospital team are necessary.

## Supporting information

S1 Table Characteristics of patients with acute myocardial infarction. (PDF)

S2 Table Factors related to intrahospital mortality among patients with acute myocardial infarction. (PDF)

S3 Table Multivariate analysis of factors related to intrahospital mortality among patients with acute myocardial infarction. (PDF)

S1 Database (XLS)

## Abbreviations

AMI: acute myocardial infarction
CRP: C-reactive protein
HR: hazard ratio
IG: Immature granulocytes
KILLIP score: risk of in-hospital death proposed by Killip and. Kimball
MPV: mean platelet volume
NLR: neutrophil to lymphocyte ratio
NRBCs: nucleated red blood cells
PDW: platelet distribution width
PROCAPE: Pernambuco Cardiac Emergency Hospital
RDW: red cell distribution width
ROC: receiver operating characteristic
STEMI: with ST elevation myocardial infarction
Non-STEMI: with non-ST elevation myocardial infarction
TIMI risk: thrombolysis in myocardial infarction risk
TNT: troponin T
WBC: white blood cell count
