# Supplementary material for: Prognostic value of hematological parameters in patients with acute myocardial infarction: Intrahospital outcomes
Source: PLoS One. 2018 Apr 18;13(4):e0194897. doi: 10.1371/journal.pone.0194897 (PMC5905886; doi:10.1371/journal.pone.0194897)
Supplement: S1 Table — (PDF) [file pone.0194897.s001.pdf]

Table 1. Characteristics of patients with acute myocardial infarction

Characteristics	Statistics
**Age** (mean ± SD)	64.2 ± 12.8
**Gender:** Male	287 (61.6%)
**Risk factors**	
Systemic arterial hypertension	335 (71.9%)
Diabetes mellitus	173 (37.1%)
Kidney disease	38 (8.2%)
Family history of coronary artery disease	220 (47.2%)
Dyslipidemia	178 (38.2%)
Depression	50 (10.7%)
Smoking	194 (41.6%)
Sedentary lifestyle	232 (49.8%)
**Acute myocardial infarction**	
**STEMI**	326 (70.0%)
**Killip score**	
Killip I and II (low risk)	298 (91.4%)
Killip III and IV (high risk)	28 (8.6%)
**Non-STEMI**	140 (30.0%)
**TIMI Risk**	
0 to 3 (low risk)	40 (28.9%)
4 to 7 (high risk)	98 (71.1%)
**Red cells**	4.40 ± 0.62
**Hemoglobin**	13.0 ± 2.00
**Hematocrit**	38.5 ± 5.22
**NRBC**	
Presence (≥1)	42 (9.1%)
Absence (0)	421 (89.9%)
**NRBC Maximum**	
Zero	421 (89.9%)
1 to 100	10 (2.2%)
101 to 200	5 (1.1%)
> 200	27 (5.8%)
**Leukocytes**	10.5 (8.4, 12.8)
**NLR**	3.71 (2.38, 5.72)
**CRP**	36.7 (11.6, 86.6)
**Platelets**	231 (195.7,278)
**MPV**	10.9 ± 0.9
**IG%**	0.3 (0.22, 0.45)
**TNT**	1.87 (0.44, 4.39)
**RDW SD**	43.2 (41.1, 45.4)
**RDW CV**	13.5 (12.9,14.2)

Abbreviations: STEMI: with ST elevation myocardial infarction; non-STEMI: with non-ST elevation myocardial Infarction; NRBC: nucleated red blood cells; NLR: neutrophil to lymphocyte ratio; CRP: C-reactive protein;

MPV: mean platelet volume; IG: immature granulocytes; TNT: troponin T; RDW SD: Red Blood Cell Distribution Width measured by Standard Deviation; RDW CV: Red Blood Cell Distribution Width measured by Variation Coefficient
