# Supplementary material for: Prognostic value of hematological parameters in patients with acute myocardial infarction: Intrahospital outcomes
Source: PLoS One. 2018 Apr 18;13(4):e0194897. doi: 10.1371/journal.pone.0194897 (PMC5905886; doi:10.1371/journal.pone.0194897)
Supplement: S2 Table — (PDF) [file pone.0194897.s002.pdf]

Table 2. Factors related to intrahospital mortality among patients with acute myocardial infarction.

Factors	Death of numbers	Death Risk	
		HR (95% CI)	p-value
**Age**			
<65 years	14	Reference	-
≥ 65 years	41	2.52 (1.37 - 6.65)	0.003
**Sex**			
Female	21	Reference	-
Male	34	1.20 (0.69 - 2.09)	0.517
**Risk factors**			
Systemic arterial hypertension	45	1.48 (0.75 - 2.95)	0.261
Diabetes mellitus	27	1.41 (0.82 - 2.40)	0.210
Kidney disease	5	1.01 (0.40 - 2.52)	0.991
Family history of coronary disease	12	0.35 (0.19 - 0.67)	0.002
Dyslipidemia	18	0.64 (0.36 - 1.13)	0.124
Depression	1	0.19 (0.03 - 1.36)	0.098
Smoking	20	1.03 (0.59 - 1.80)	0.911
Sedentary lifestyle	32	1.23 (0.72 - 2.12)	0.442
**Laboratory Measures**			
Erythrocyte[a]	-	0.41 (0.25 - 0.68)	0.001
Hemoglobin[a]	-	0.83 (0.71 - 0.98)	0.024
Hematocrit[a]	-	0.93 (0.87- 0.98)	0.018
Leukocytes (>10.5)[b]	45	4.57 (2.30 - 9.09)	0.000
CRP (>36.7)[b]	41	3.55 (0.46 - 3.88)	0.002
Platelets (>231)[b]	8	6.93 (3.21 - 14.9)	0.000
IG% (>0.3)[b]	49	6.09 (2.59 - 14.3)	0.000
TNT (>1.87)[b]	37	3.03 (1.69 - 5.42)	0.000
RDW SD (>43.2)[b]	43	3.56 (1.83 - 6.92)	0.000
RDW CV (>13.5)[b]	43	3.15 (1.61 - 6.12)	0.001
NLR:			
<3.7	4	Reference	-
≥ 3.7	51	11.3 (4.06 - 31.2)	0.000
NRBC:			
Absence (0)	26	Reference	-
Presence (≥1)	29	5.65 (3.23 - 9.88)	<0.001
MVP:			
<10,4	6	Reference	-
≥ 10.4	49	4.46 (1.78 - 11.2)	0.001

[a] Decreased risk with the increase of one unit of the laboratory marker

[b] Risk for values above the median
