# Supplementary material for: Prognostic value of hematological parameters in patients with acute myocardial infarction: Intrahospital outcomes
Source: PLoS One. 2018 Apr 18;13(4):e0194897. doi: 10.1371/journal.pone.0194897 (PMC5905886; doi:10.1371/journal.pone.0194897)
Supplement: S3 Table — (PDF) [file pone.0194897.s003.pdf]

Table 3. **Multivariate analysis of factors related to intrahospital mortality among patients with acute myocardial infarction.**

**Factors**	**Death Risk**	
	**HR (95% CI)**	**p-value**
**Age**		
<65 years	Reference	-
≥ 65 years	1.88 (1.02 - 3.49)	0.043
**Laboratory Measures**		
Leukocytes (> 10.5)[a]	2.01 (0.97 - 4.17)	0.059
TNT (> 1.87)[a]	1.76 (0.98 - 3.16)	0.057
NLR:		
<3.7	Reference	-
≥ 3.7	5.02 (1.68 - 15.0)	0.004
NRBC:		
Absence (0)	Reference	-
Presence (≥ 1)	2.42 (1.35 - 4.36)	0.003
MVP:		
<10,4	Reference	-
≥ 10.4	2.97 (1.15 - 7.67)	0.024

[a] Risk for values above the median
